# Rehabilitation guidelines after autograft anterior cruciate ligament reconstruction need more graft‐specific exercise recommendations—A scoping review

**DOI:** 10.1002/ksa.12666

**Published:** 2025-04-03

**Authors:** Kristín Briem, Mette Kreutzfeldt Zebis, Bjarki Þór Haraldsson, Jesper Bencke, Linda Fernandes

**Affiliations:** ^1^ Department of Physical Therapy Faculty of Medicine, University of Iceland Reykjavík Iceland; ^2^ Department of Midwifery, Physiotherapy, Occupational Therapy, and Psychomotor Therapy Faculty of Health, University College Copenhagen Copenhagen Denmark; ^3^ Institute of Sports Medicine Copenhagen, Bispebjerg and Frederiksberg Hospital, Copenhagen University Hospital Copenhagen Denmark; ^4^ Copenhagen University Hospital, Amager‐Hvidovre Copenhagen Denmark

**Keywords:** ACL reconstruction, patellar tendon, physical therapy, quadriceps tendon, semitendinosus

## Abstract

**Purpose:**

Autografts for anterior cruciate ligament reconstruction (ACLR) are primarily harvested from the quadriceps, patellar, and semitendinosus tendons. The purpose of this scoping review was to describe available recommendations for exercise‐based rehabilitation following primary ACLR with a quadriceps tendon (QT), semitendinosus tendon (ST), or bone‐patellar‐tendon‐bone (BPTB) autograft and determine whether these recommendations included graft‐specific clinical practice guidelines (CPGs).

**Methods:**

A search was conducted via three electronic databases, using variations of three main strings: ‘anterior cruciate ligament reconstruction’, ‘rehabilitation’ and ‘guideline’. To be considered eligible, publications had to be published between 2014 and 2024, target patients 16 or older, and include exercise‐based recommendations for rehabilitation after primary ACLR using QT, BPTB or ST autografts. Identified papers were screened for title, abstract and full text in accordance with a pre‐registered protocol, with specific inclusion and exclusion criteria. Charting of data found within eligible publications was done according to their overall exercise‐based content, as well as any graft‐specific considerations.

**Results:**

A total of 1083 publications were imported for screening, but after the removal of duplicates and subsequent screening of titles, abstracts and 98 full texts, 17 remained for inclusion. The timeline and implementation of different exercise modalities involving knee joint loading varied during the earliest phases of rehabilitation. Sixteen papers included one or more graft‐specific considerations, the majority of which focused on protecting the graft and/or considerations relating to the BPTB harvest site. Few focused on the ST or QT harvest sites, and only one publication provided guidelines that considered all three autografts.

**Conclusion:**

CPGs providing exercise recommendations and post‐surgical considerations for all three autograft types are needed. These would provide a comprehensive and valuable resource for clinicians to plan rehabilitation for patients who have undergone ACLR, mindful of graft choice and surgical procedure.

**Level of Evidence:**

Level V.

**Abbreviations**
ACL: anterior cruciate ligament
ACLR: anterior cruciate ligament reconstruction
BPTB: bone‐patellar‐tendon‐bone
CKC: closed kinetic chain
CPG: clinical practice guideline
NMT: neuromuscular training
OKC: open kinetic chain
PRISMA: preferred reporting items for systematic reviews and meta‐analyses
PRISMA‐ScR: PRISMA extension for scoping reviews
QT: quadriceps tendon
ROM: range of motion
SR: systematic review
ST: semitendinosus
WB: weight‐bearing

## BACKGROUND

Anterior cruciate ligament (ACL) reconstruction (ACLR) in athletes typically involves the use of an autologous graft harvested from the ipsilateral semitendinosus, patellar, or quadriceps tendon (QT) [2, 3, 66]. Following ACLR, the influence of graft selection on outcomes, such as pain, joint stability, muscle strength and graft failure rates, has been investigated in numerous systematic reviews (SRs) and meta‐analyses [10, 13, 14, 41, 51, 68, 75, 87]. However, few address post‐surgical rehabilitation, despite procedure‐dependent influence on the donor site's mechanical integrity and function [18, 29, 31, 36, 67] and diverse fixation techniques [66]. Harvesting the bone‐patellar‐tendon‐bone (BPTB) and QT grafts impacts the tendon and myofascial structure leading to persistent quadriceps strength deficits [27], but the tissue's continuum is intact. In contrast, harvesting the semitendinosus tendon (ST) may reduce quadriceps deficits [27], but has direct mechanical impact on hamstrings function due to subsequent ST atrophy and retraction, regardless of tissue regeneration [17, 26, 39]. Grafts must undergo osseous integration and ligamentization [3], where graft type and fixation influence the timeline for each process [58, 66]. This is an important consideration when introducing loads to the knee.

A recent European survey found that surgeons considered graft choice and concomitant injury for degree of weight‐bearing (WB), bracing and range of motion (ROM), but that guidelines for rehabilitation were generic [49]. Similarly, clinical practice guidelines (CPGs) for post‐ACLR rehabilitation seldom provide detailed recommendations based on graft type or fixation. Most CPGs provide some functional milestones (e.g., ROM, muscle function and movement patterns) [33, 49, 54, 65] but limited graft‐specific considerations, mainly in the early post‐operative phase [65]. Recent publications on post‐ACLR rehabilitation have contrasted a limited number of interventions (e.g., delayed vs. accelerated; open vs. closed chain) [1, 21, 56, 60], rather than evaluating comprehensive rehabilitation protocols, or seeking evidence‐based CPGs for rehabilitation after ACLR with specific concomitant injuries [12, 77]. Their assessment of the quality of the studies reviewed highlights a lack of evidence [1, 12, 56, 77] which is why any recommendations relating to knee joint loading tend to be cautious. While some CPGs incorporate expert opinion from panels [15, 22], few rely on research that specifically examines tissue tolerance to loading. Graft types present unique challenges influencing rehabilitation, but without tailored guidelines, therapists rely on generic protocols and clinical judgment, which may increase variability in outcomes and suboptimal recovery.

Scoping reviews map existing knowledge and identify gaps, providing a comprehensive overview to guide future studies and improve clinical practices [63]. The approach may ultimately improve standards in orthopaedics, sports medicine, and rehabilitation, which are currently lacking in this area. Therefore, a scoping review was conducted to describe recommendations that include specific exercise‐based and graft‐specific considerations for rehabilitation following primary ACLR with QT, ST or BPTB autografts. By charting available recommendations and restrictions, evidence gaps will be identified while providing an extensive overview of available protocols and their supporting evidence.

## METHODS

This scoping review adhered to guidelines relevant to CPGs [35, 52, 79], using the preferred reporting items for systematic reviews and meta‐analyses (PRISMA) extension for scoping reviews (PRISMA‐ScR) [61, 79].

### Protocol and registration

A protocol for search methods and data extraction was documented and registered with the Open Science Framework (osf.io) prior to the search.

### Eligibility criteria

To help formulate the question and criteria for inclusion, the PICAR framework was used (P = population or clinical indication or condition, I = intervention, C = comparator or content, A = attributes for eligibility, R = recommendation characteristics) [35].

### Population

Recommendations concerning persons aged 16 or older who had undergone primary ACLR using any of the three targeted autografts (BPTB, QT and ST) were considered for inclusion. As allografts lack a relevant ipsilateral donor site influencing rehabilitation, publications primarily studying allografts were excluded. Publications, where participants had undergone major surgical procedures due to concomitant injuries or secondary ACLR, were excluded as these would significantly influence their rehabilitation approach. Publications focusing on children were excluded due to differences in pediatric surgical and rehabilitation approaches.

### Intervention

Studies of interest included recommendations about any exercise therapy that loaded the knee joint and surrounding tissues. Exercises involving knee joint loading were defined as those involving WB and/or specific muscle activation introducing forces acting on the graft donor site or the graft and its fixation. Passive rehabilitation modalities such as passive knee joint ROM were excluded because of limited loads onto the graft or donor site, whereas specific stretching exercises were considered, as their goal involves strain to the muscle/tendon complex. The window of interest was during the first 9 months after surgery. Recommendations for pre‐ or non‐surgical interventions were excluded.

### Comparator

All comparators were of interest, but any guidelines on methodology or tests to use when assessing function, or primarily surgical recommendations were excluded.

### Attributes

To be eligible for inclusion, peer‐reviewed international publications could be any of the following: primary research, SRs with or without meta‐analysis, scoping or narrative reviews, rehabilitation protocols/guidelines, clinical commentaries and technical notes, including rehabilitation guidelines after primary ACLR. Case studies, surveys, secondary publications, conference abstracts, opinion pieces, pamphlets for patients, and magazine or newspaper articles were excluded. To ensure that included publications were presenting recommendations based on current surgical methods and clinical knowledge, publication date limits were set in 2014 and later. The languages included were: Danish, English, German, Icelandic, Italian, Norwegian and Swedish.

### Recommendation characteristics

To be eligible, the publication had to include at least one clear, standalone statement within the main text, in tables, algorithms or in Supporting Information S1 [35]. No quality score cut‐off was applied.

### Information sources and search strategy

**Figure 1:**
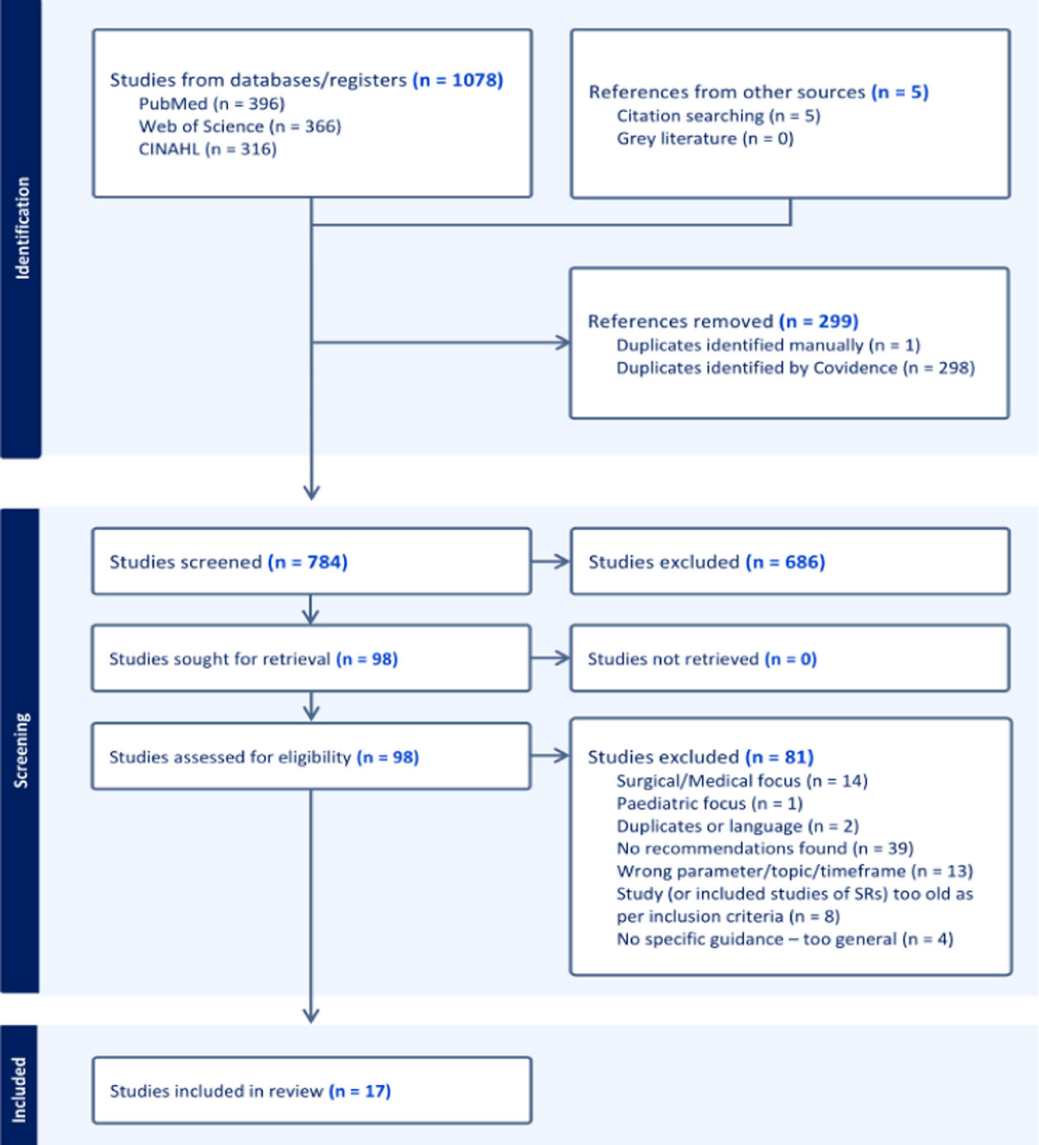
Flow chart of search, screening and full‐text evaluation process.

The initial search was conducted on 31 October 2023, using the PubMed/MEDLINE, Clarivate/Web of Science and EBSCO (CINAHL) electronic databases, and updated on 25 September 2024, to identify any new publications. The full electronic search strategies are presented in supplementary documentation and include variations of subject headings and keywords of the following three focus areas: #1 ‘anterior cruciate ligament reconstruction’; #2 ‘rehabilitation’; #3 ‘guideline’. The Boolean ‘OR’ operator term was used between terms within the same search string, while the ‘AND’ operator term was used to combine the three. Initial screening of titles and abstracts was independently performed by two persons (K.B. and L.F.), with any discrepancies addressed by a third reviewer (MKZ) and a final consensus made in a meeting of all three reviewers. The same process was used for full‐text review. All identified publications from each search were downloaded and imported (RIS format) into separate EndNote libraries. These were exported into Covidence SR software (Veritas Health Innovation), where duplicates were removed and the site used for further screening (Figure 1).

### Data charting process

Charting of data was aided by three consecutive tables. First, charting of standard information was performed (authors, year of publication, study design, study aim; Table 1). Second, charting of exercise descriptors was conducted with specifics about timing, exercise characteristics, ROM, loading and progression strategies (Table 2). Information was categorized and collated into distinct rehabilitation phases based on the number of weeks post‐ACLR: Immediate (0–2), Early (2–3), Intermediate I (4–6), Intermediate II (7–9), Transition I (10–12), Transition II (13–16) and Late (17+). Third, charting of graft‐specific restrictions or considerations relating to rehabilitation activities was done by graft type and organized to reflect the rationale for the recommendation given (e.g., due to loading of the graft and/or the harvest site; Table 3).

**Table 1:** Included papers with first author, year of publication, type of study and aims.

Authors	Year	Type of publication	Aims
Brinlee et al. [4]	2022	Clinical review	Revisit and update ACLR rehab guidelines, validate milestones
Buckthorpe et al. [8]	2019	Clinical commentary	Discuss strategies to optimize quadriceps recovery after ACLR
Buckthorpe and Della Villa [6]	2020	Review	Provide recommendations on mid‐stage rehab after ACLR
Buckthorpe et al. [5]	2021	Review	Translate research on hams function, apply to ACLR rehab
Buckthorpe et al. [7]	2023	Review	Provide recommendations on early‐stage rehab after ACLR
Diermeier et al. [16]	2020	Narrative review	Not stated
Hunnicutt et al. [29]	2020	Technical note	Describe implications of QT graft for early care after ACLR
Kotsifaki et al. [40]	2023	Clinical guideline	Inform clinical practice after ACLR
Leung et al. [43]	2023	Narrative review	Discuss post‐operative ACLR rehab based on graft type
Malempati et al. [48]	2015	Commentary	Provide basis for formulating athletes' rehab pre‐/post‐ACLR
Panariello et al. [57]	2017	Commentary	Propose an effective rehab template considering healing, strength and conditioning
Peebles et al. [59]	2019	Review	Not stated
Smith et al. [72]	2014	Commentary	Provide knowledge/principles for nurses on ACL tears
Solie et al. [74]	2023	Commentary	Present procedure‐specific considerations for ACLR with QT
van Melick et al. [80]	2016	Clinical guideline	Describe rehab after ACLR based on an SR and consensus
Wilk and Arrigo [83]	2017	Commentary	Provide a scientific basis for the rationale for rehab after ACLR
Wright et al. [84]	2015	Clinical guideline	Review available evidence and provide guidelines after ACLR

*Note*: Type of publication is listed as per the journal's provision or according to the authors' statement.

Abbreviations: ACLR, anterior cruciate ligament reconstruction; hams, hamstrings; QT, quadriceps tendon; rehab, rehabilitation; SR, systematic review.

Synthesis of results: Charted data were organized into tables and graphs for the presentation of results, with all authors approving their final format. Exercise recommendations were organized by: (1) timing (within each phase), including details regarding the timespan of each recommendation; (2) exercise modalities based on their focus: WB, strength training using open kinetic chain (OKC) or closed kinetic chain (CKC) approaches; neuromuscular training (NMT), including balance and agility; more demanding/complex exercise such as running progression, plyometrics and sport‐specific exercises. Some overlap was expected, e.g. regarding gait training, which was categorized as a WB exercise (with full‐ or partial‐WB or WB as tolerated) even though this may also be considered to include elements of NMT. Likewise, a CKC exercise includes WB and elements of NMT but was categorized as a CKC exercise when the intent was mainly to achieve strengthening; (3) degree and manipulation of ROM and loading (limitations and progression) were organized within a specific column for that information. Similarly, graft‐specific information was organized by: (1) time for which the restriction or consideration was valid; (2) rationale given by authors about forces acting on the graft/fixation or harvest site; (3) descriptions of the limitations or promotions identified for specific exercises. Recommended exercises in Table 2 identified with a symbol, indicated that considerations should be checked for in Table 3.

## RESULTS

A total of 1083 publications were imported for screening but only 17 presented exercise‐specific recommendations (Figure 1). An overview of year and type of publications, their authors and stated aims are shown in Table 1.

### Characteristics of publications

**Figure 2:**
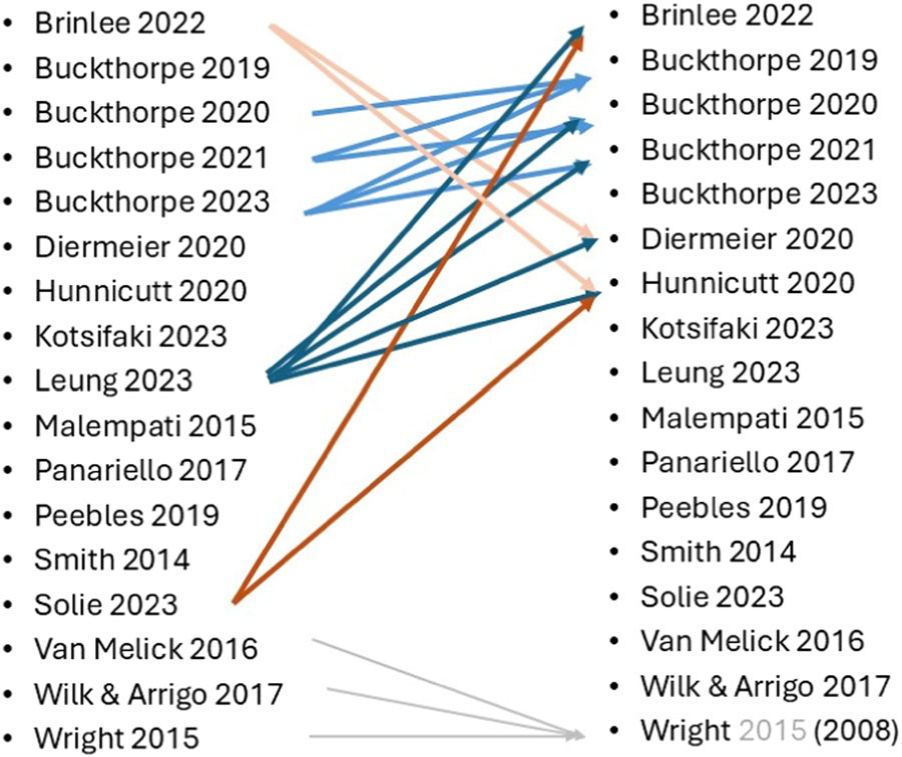
Inter‐referencing between the eligible publications, showing which authors (left column) are referencing others (right column). The publication year for Wright et al. is in grayscale as the referenced paper was an older version of their guidelines.

One of the included publications presented a systematic search to underpin their recommendations [80], while the remaining 16 publications were Levels IV to V evidence. One used available clinic records to inform recommendations [4]. Upon inspection, some inter‐publication referencing was found among the more recent publications, which should be considered when weighing the recommendations made (Figure 2). Four of the identified publications originate from the same research group and include overlapping information and some self‐citation in support of recommendations [5, 6, 7, 8]. Three others [4, 43, 74] referenced a total of five of the other papers [4, 5, 6, 16, 29] to support clinical statements made. One of the eligible papers [84] referenced an older CPG from their own group [85, 86], which was also referenced by two other of the included publications [80, 83].

### Main exercise modalities through phases

In short, the immediate phase (w. 0–2) was dominated by a pool of exercises to regain WB, active ROM, and muscle activation (Table 2). During the early phase (w. 2–3), OKC isometric exercises for quadriceps and hamstring muscles were frequently recommended, along with the introduction of dynamic OKC and CKC. The intermediate phase I (w. 4–6) was dominated by dynamic OKC and CKC exercises. Transition from gait retraining to cardiovascular modalities was seen from w. 4 to 6, as were specific NMT exercises. The combination of OKC, CKC and NMT was found through the intermediate II (w. 7–9) and transition I (w. 10–12) phases, with running re‐education beginning from w. 10. During transition II (w. 13–16) and late phase (w. 17+), the exercise modalities shift towards more gym‐based programmes. Few studies have recommended aquatic exercises or stretching compared to other exercise modalities.

### Exercises progression strategies

Considerable heterogeneity was found regarding initiation and timing of progression of the various exercise modalities. For example, during w. 4–6 recommendations to ROM varied between 90–50° and 90–0° for OKC knee extensions and between 0–30° and 0–100° for CKC exercise, without the studies providing graft‐specific or other rationales (Table 2). Progression generally involved common principles of biomechanics and strength training, such as more loads during WB, strengthening through a greater ROM, and greater resistance. Eventually, greater emphasis was placed on more complex tasks, plyometrics, medium to heavy load strength training (both OKC and CKC), and cardiovascular fitness and sport‐specific drills (Table 2).

**Table 2:** Recommended exercises following primary ACLR.

Phase	Exercise modality	Exercise description	Criteria and progression		Time (if specified)
w. 0–2 immediate	WB	Gait retraining [4, 5, 7, 29, 40, 48, 57, 59, 80, 83, 84]	ROM	If full ext [57], >60 flex [57] and SLR no lag [57, 59]	w. 0–4 [48, 57]
			Load	WBAT [4, 29, 40, 48, 80, 83, 84], PWB [59, 72]	
	ROM	AROM [16, 40, 57, 59, 83],^†^ AA flex [57], seated AA flex [84]	ROM	90‐0 [16],^†^ no ROM limitations [59], reach 90 [83]	By d. 5 [83]
			Load	3–4 sets of 20+ reps [16],^†^ progress to flex against gravity [57, 83]	w. 0–4 [16]
		Wall/heel slides [48, 57, 59, 72, 80, 83, 84]	ROM	Gravity‐assisted knee flex [72]	
			Load	Slide into flex, use AA ext [59]	
		Stationary bike [48, 59, 84]	ROM	Partial revolution [59, 84], progression of ROM [48]	w. 0–4 [48]
	OKC^*†^	Quads set/isometric/SLR [4, 7, 8, 16, 29, 40, 48, 57, 59, 72, 74, 80, 83, 84]^*†^	ROM	At 90‐60 [7, 83],* holds of 5 × 45 s, 1–2/day [7]	w. 0–4 [16, 74]^†^
			Load	Low load [8, 84]	
		LAQ [4, 7, 43], SAQ [16, 29, 83, 84]^†^	ROM	Through 90‐0 [4, 7, 43], 90‐40 [83], 90‐60 [16]^†^	w. 1–6 [84]
			Load	Light ankle cuff weight [4, 43, 84] to 3 kg [5], heavy cuff weight 2–3 sets of 15+ reps [16]^†^	w. 0–4 [16]
		Hams isometric [59, 84], co‐contraction [5, 84]^‡^	Load	Static, low volume [5]^‡^	
		Prone knee flex [59, 84], isometric knee flex [5]^‡^			
	CKC^†^	TKE [4, 29, 48, 57, 74, 84]^†^	Load	2‐4 sets of 10‐20 reps [74], Theraband [57, 74]	w. 0–4 [57, 74] w. 0–6 [29]^†^
		Mini squats, weight shifts [83]			
	NMT	During OKC/CKC [83]			d. 4–7 [83]
	Stretch	Hamstring stretch [48, 83], rectus femoris [16]^†^	ROM	Manipulate hip ROM [16]^†^	
w. 2–3 early	WB	Gait retraining [29, 48, 57, 83], stair stepper [83]	ROM	With full knee ext [29]	
			Load	Progressive loading [48, 57], full WB [83]	
	ROM	Ecc cycle ergometer or stepper [40], bike [59, 80, 83]	ROM	20‐60 [40], partial revolution [59], flex >100 [80]	
	OKC^*†‡^	Isometric ext [4, 8, 29, 43, 59, 72, 74, 84], SLR [29, 57, 59, 74, 84]^†*^	ROM	At multiple hip [43]^*†^ and knee [4, 43, 72]^*†^ angles	
			Load	Increase light [84] load [29]	
		Isotonic/‐kinetic ext [4, 29, 43, 74, 83, 84]	ROM	LAQ [4, 74], SAQ [29, 74], 90‐40 [84] ecc, 40‐100 [83]	w. 2–6 [84]
			Load	Add weight if no lag [29], add 1 lb/w [83], light cuff [4]	
		Hams isometric [5]^‡^/curls [4, 5, 40, 48, 57, 59, 83, 84]^‡^	ROM	Short/med muscle length [5]^‡^	w. 1–2 [5]^‡^
			Load	Low intensity, through hip > knee [5], standing [57]	
	CKC	CKC [4, 7, 29, 40, 43, 48, 57, 59, 80, 83, 84], weight shift [4], TKE [48, 57], air/mini squats [4, 7, 29, 48, 83, 84], split‐squat [74], wall sit [4, 48], leg press [40, 48, 83, 84], step up [7, 84], mini‐lunge [48], front/side lunges, step down, lateral step‐over [83], bridging [59]	ROM	Through 0‐45 [40], 0‐40 [83], 45‐60 [48], 0‐60 [80, 83]	w. 0–6 [59] from w. 2 [80], w. 2–6 [84], w. 3 [40]
			Load	In water [7], progress bridging from DL to SL [59], con CKC [80], progress to squats on tilt‐board [83], SL [4, 84]	
				Start ecc. CKC [80]	w. 3 [80]
	NMT	Balance [7, 80, 83, 84]			
	Aquatic	Aquatic gait [7, 83], CKC [7, 40, 59, 83]			From w. 2 t 3 [5]/3 [40]
	Stretch	Stretches for quads [7, 29, 84]/ext [5], hams [84]	ROM	Via hip and knee (RF) [29]	w. 2–6 [84]
w. 4–6 intermediate I	WB/ROM/Cardio	Bike [4, 8, 48, 59, 72, 83], treadmill walking [83], unloaded [83] gait [6, 29, 72], stairmaster [4, 43, 48]	ROM	If ROM > 110 [72]	From w. 4 [43]/5 [72]/6 [59]
			Load	Progress by greater duration [59]	
	OKC^†‡^	Isometric ext [72, 83], TKE standing [74]	Load	Progress isometric [83], theraband [74]	w. 4–8 [74], w. 5–11 [72]
		Knee ext [4, 6, 8, 16, 29, 40, 43, 72, 74, 80, 83]^††‡^	ROM	90‐60 [16],^†^ 90‐50 [6], 90‐45 [40, 48, 72, 80],^‡^ 90‐40 [83], 90‐30 > 20 [80], 90‐0 [29], LAQ [74]	w. 4–8 [74] w. 5–8 [16]
			Load	Manipulate speed/ROM [4], reps/weight [29, 40, 48, 80], 15–25 RM (3 s up/down) [7], 3–4 sets to fatigue [74], increase intensity [7], 3–4 sets of 15+ reps [16],^†^ cable column for RF [16]^†^	
		LAQ [16]^†^	ROM	90‐0 [16]^†^	
			Load	Cuff weight, 2–3 sets of 20+ reps [16]^†^	w. 5–8 [16]
		Hamstrings curls/standing flex [4, 5, 48, 83]^‡^	Load	Low/mod intensity [5]^‡^	
		Train hamstrings via hip and knee (seated/prone) [5]^‡^	Load	Mod/high volume [5]^‡^	
		Isometric hamstrings [5, 6, 43]^‡^	ROM	At 90/60/0 [43]	w. (5)6 [8]
	CKC	CKC [4, 6, 7, 8, 16, 29, 48, 57, 59, 72, 80], bridges [4, 59], deadlifts [6, 48, 57], DL [74], SL [4, 6, 16, 48], BW [29, 57, 59, 72, 83], overhead [57], back [57], split [16] squats, step up/down [4, 6, 48], leg press [4, 6, 48, 57, 59, 83], lateral [83], lunges [4, 6, 29]	ROM	0‐60 [74], 0‐45/60, then 0‐90 [48], 0‐30 [72], to >90 [57], hip dominant [59], 0‐90 [59], 0‐100 [83]	w. 4–8 [74] w. 4–12 [8], w. 5–8 [57], w. 6–12 [59]
			Load	3 sets of 10–15 reps [57], 12–20 RM [6, 8], increase resistance [57], add weight to squat [29], depth in squat to 90 flex [57], ROM (w. 8) [72], DL to SL [29, 48, 57, 59], ecc to con [57], low load, high reps, limited rest [59], low to mod load [74], kettlebells [16]	
	Aquatic	Aquatics [59, 72, 83]			
		Running backwards [83]			w. 4 [83]
		Running forwards [83]			w. 6 [83]
	NMT	Neuromuscular/balance/perturbation [4, 48, 59, 80, 83, 84]			
		SL balance [59, 84], SL drills [16],^†^ squats/ball throws on tilt board [83]			w. 2–6 [84], from w. 6 [16, 59]
w. 7–9 intermediate II	WB/Cardio	Stationary bike [6, 59, 83], walking program [83], Stairmaster/elliptical [72, 83]			From w. 8 [72]
	OKC^†‡^	Knee ext [4, 5, 8, 16, 40, 43, 74, 80, 84]^‡,†^	ROM	90‐60 [16],^†^ 90‐45 [74],^†^ 90‐10 to 0 [80], 90‐0 [16, 84]^†^	w. 7–8 [80], w. 7–12 [84]
			Load	SAQ resistance 3–4 sets of 8–12 reps [16],^†^ SL con/ecc 3–4 sets to fatigue [74], LAQ cuff 3–4 sets 15+ reps [16]^†^	w. 8–12 [16, 74]
		Isokinetic ext [83]	ROM	90‐40 [83]	
			Load	At 120/240°/s [83]	
		Con/ecc ext [4, 40, 80]	ROM	Through 90‐0 [4]	
			Load	At 60%–75% of 1RM [4]	
		Isom [6],^‡^ dynam [4, 6, 43, 84],^‡^ ecc [43], knee flexion	ROM	0‐90 [43]	w. 8–12 [43]
			Load	Progressively increase load [43], tempo 1:1:5 (con:isometric:ecc) [43]	
		Hams flex [84] w. tibial rotation6^‡^			w. 7–12 [84]
	CKC^‡^	CKC [4, 5, 8, 40, 43, 80, 84], squats [5, 57, 59, 72, 80, 83, 84], wall‐squat [74], split‐squat [74], lunges [57, 72, 80, 83, 84], SL roman deadlift [59], leg press [4, 59, 83, 84], Nordic hams [6]^‡^	ROM	60‐90 [74] (wall), 0‐60 [74] (split), 0‐90 [59], full ROM [72], 0/40‐100 [83]	w. 8–12 [74], w. 6–12 [59], from w. 8 [72], w. 8 [80]
			Load	DL to SL and progress intensity [6, 57, 59], SL variation [4, 83], weight [4], progress as with OKC [4, 8], 3–4 × 45–90 s [74], 3–4 sets of 10–15 reps [74]	
	Aquatic	Pool running [6]			
	NMT^‡^	Neuromuscular/balance/perturbation [4, 72, 83, 84]	Load	Multi‐task [4], ‐surfaces [84], add perturbation [4, 84]	w. 5–11 [72], w. 7–12 [84]
		Functional exercises [5, 43, 84],^‡^ ecc [5] via hip and knee [5, 43], shuttle run/step up and down/hopping [84]	Load	Mod loads (6–12RM), low > mod [5]^‡^ explosivity [5], progress hopping from DL to SL	w. 7–12 [84]
	Plyometric	Plyometric [83]	Load	Plyometric leg press [83]	w. 8–10 [83]
	Stretch	Flexibility training (quads/hams) [72]			w. 5–11 [72]
w. 10–12 transition I	WB/Cardio	Running re‐education [6, 72, 80, 83]			From w. 10 to 18 [83], from w. 12 [72]
	OKC^†‡^	Burst test for quadriceps [4]			w. 12 [4]
		Knee extension [4, 8, 16, 74]^†^	Load	6–10 sets of 8–12RM [8], 2–3 sets 20+ reps (90‐0) [16], 3–4 sets of 6–10 reps (90‐45) [16], 3–4 sets of 15–20RM [74]	w. 9–12 [8], w. 12–16 [16, 74]
		Isokinetics (con) [83] ext/flex	ROM	90‐40 [83]	
			Load	At 180/300°/s [83]	
		Con/ecc overload [4]			
		Hams progression [6]^‡^	Load	At 8–12RM [6]	
	CKC	Functional CKC training [6, 57, 59], squat [8], Olympic lift [57], leg‐press [74], split‐squat [16, 74]	ROM	60‐90 [74] (split‐squat)	
			Load	Manipulate time [59], 6–10 sets of 8–12RM [8], 3–4 sets of 10–20RM [74]	w. 9–16 [57], w. 12–16 [74]
	NMT	Balance [59, 80, 83]	Load	Kettlebell [57], manipulate DL to SL [83] and time [59]	
	Plyometric^†‡^	Plyometrics [4, 16, 40]^‡†^/agility [40]	Load	Full power plyometric may be neglected [16]	From w. 12 [4, 16]
	Stretch	Stretching drills [83]			
	Sport	On‐field rehab, ball striking [16]^†^			From w. 12^D†^
w. 13–16 transition II	Cardio	Initiate jogging/running [4, 8, 48], backwards [83]			From m. 3 [48]
		Swim, elliptical, bike, stairmaster [59]			
	Strength	Gym based program [4, 8], knee ext [8, 74], leg press [5, 6, 7, 8, 29, 43, 48, 57, 59, 74], squats [8, 48, 59], split‐squat [74], Nordic hamstrings [5, 43], deadlift [5, 8, 43, 59], functional resistance training [8]	ROM	Hip dominant [59], rear‐foot elevated [74]	
			Load	Use 5–10 RM, mod. volume [8], 3–4 sets/8–12 reps manipulate weights [59], at 6–12RM [8], 6–15RM 3–4 sets to fatigue [74]	
		Aggressive strengthening [48, 84]			From m. 3 [48]
		Hams (ecc overload), vary tibial rot [43]	Load	Progress sets/reps from 2 × 5 to 3 × 12 [43]	From w. 13 [43]
		Lunges and hams curls (physioball) [43, 59]	Load	Multidirection [59]	
	Plyometric^†‡^	Plyometric [40, 48, 72, 74, 84]^†^ and lat [83] agility [5, 40, 48, 84] drills			
		Jump/hop/landing mechanics [4, 8, 48, 72]^†^	Load	DL to SL [48], multidirection [48], higher level balance progression [4, 8, 48, 72]	From w. 16 [74]^†^
	Sport	Sport‐specific drills [29, 84]	Load	At 50%–75% effort [84] progress to RTS [84]	
w. 17+ late	Strength	Con/ecc overload [4, 74], heavy barbell squat [4, 59], leg‐press [74]	Load	At 60%–80% 1RM [4], 1–5RM [74], DL up/SL down [74], heavy load [4, 8]	
		Deadlifts [4, 5, 59], kettlebell swings [4], ballistics [8, 59]		Functional RT at 3–8 RM [8]	
		Hams con/ecc/isom in lengthened positions [5]		Mod/high explosivity [5]	
		Olympic lifts [8, 59], Nordic hams [4, 5, 59]		High intensity and mod vol [5]	
	Plyometrics	Plyom [4, 5, 8, 57], agility [4]/reaction drills [59]	Load	Multiplane [4], high intensity and mod vol [5]	From m. 6 [74]
		Bound/jog/run/sprint [4, 57, 59], lunge, jump/hop [4, 59, 74]		Sport‐specific [4], manipulate reps [57], multiple planes [4], 40%–60% 1RM [74]	
	Sport	Sport‐specific training [83]			
		Unrestricted training with team [72]			From m. 6 [72]

*Note*: Graft‐specific restrictions apply for *BPTB, ^†^QT and ^‡^ST (Table 3).

Some authors recommend crutches [4, 29, 48, 59, 72, 83, 84] for 0–1 [4], 0–2 [29, 59] or 0–4 [48, 72] weeks and/or knee brace [48, 59] for 0–2 [59] or 0–4 [48] weeks during gait training for limited WB and safety. Many authors recommend augmenting strengthening by using NMES and/or BFR during the earliest phases and continuing if strength remains low.

Abbreviations: AA, active assisted; ACLR, anterior cruciate ligament reconstruction; BW, body weight; CKC, closed kinetic chain; con, concentric; DL, double leg; ecc, eccentric; ext, extension; flex, flexion; hams, hamstrings; LAQ, long arc quads; OKC, open kinetic chain; PWB, partial weight‐bearing; quads, quadriceps; RF, rectus femoris muscle; RM, repetition max; ROM, range of motion; RTS, return to sports; SAQ, short arc quads; SL, single leg; SLR, straight leg raise; TKE, terminal knee extensions; WBAT, weight‐bearing as tolerated.

### Graft‐specific restrictions and considerations

**Figure 3:**
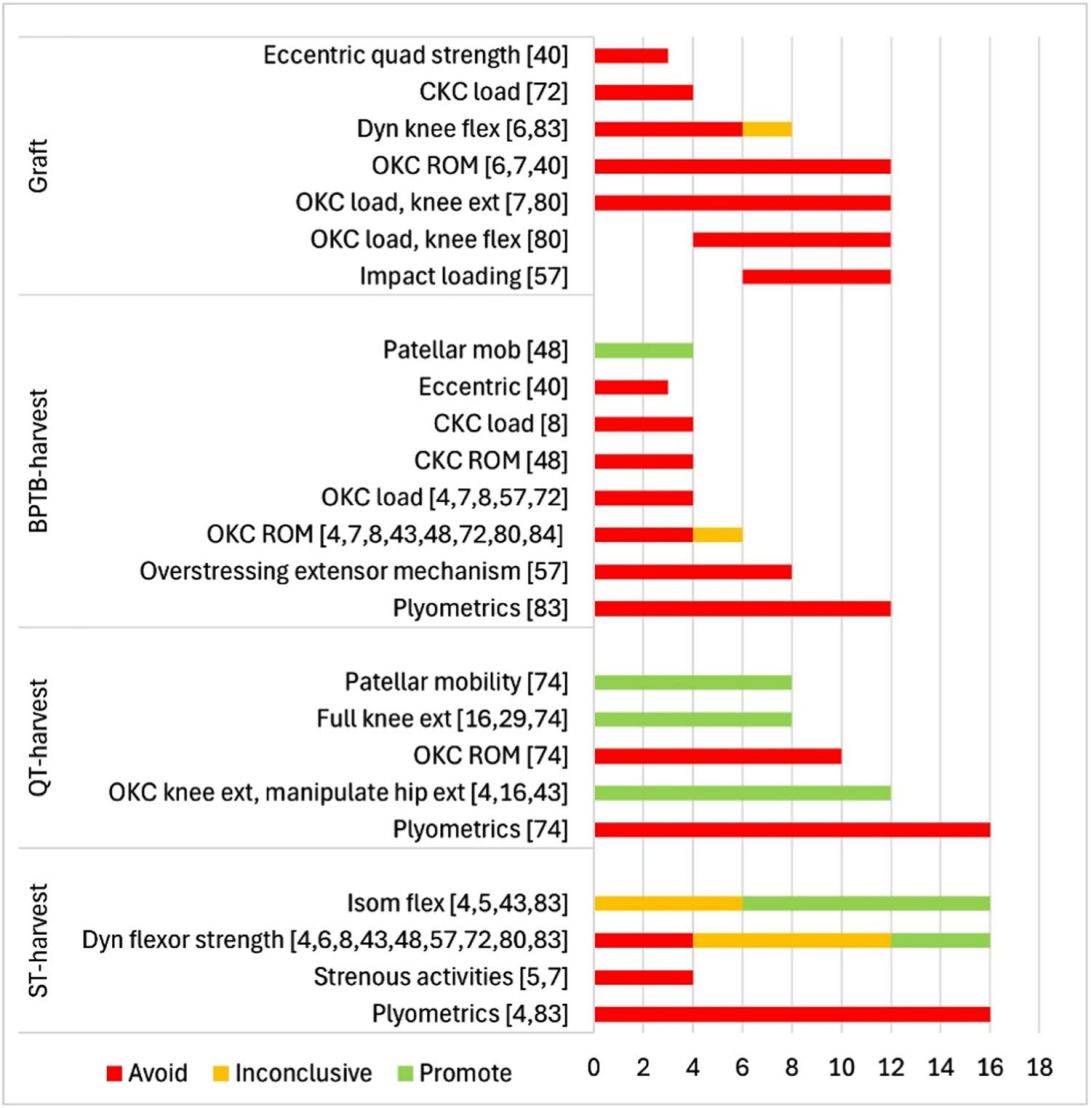
Aggregated data of graft‐specific restrictions and considerations ordered by graft and harvest sites. Exercises (*y*‐axis) and weeks from surgery (*x*‐axis). Red bars indicate avoidance or caution (limited ROM or load), yellow bars indicate different time limits between publications, and green bars indicate the promotion of an exercise. BPTB, bone‐patellar‐tendon‐bone; CKC, closed kinetic chain; Dyn, dynamic; ecc, eccentric; ext, extension; flex, flexion; isom, isometric; quad, quadriceps; OKC, open kinetic chain; QT, quadriceps tendon; ROM, range of motion; ST, semitendinosus.

Of the 17 included studies, 16 described one or more graft‐specific restrictions or considerations (Table 3). Guidelines were provided in some form from Day 1 until w. 16, most of them during the first 4 weeks (Figure 3). However, only a single study [4] included graft‐specific restrictions of both graft/fixation and the three harvest sites (BPTB, QT and ST). Nine publications provided graft‐specific restriction for a single graft type, five of which were for BPTB [8, 40, 72, 83, 84], three for QT [16, 29, 74], and one focused on ST graft only [5]. Rationale provided for considering the ST graft and harvest site focused on allowing for healing and, thus, for the protection of the graft or harvest site. Rationale provided for considerations to the BTPB harvest site on the other hand, focused on avoiding anterior knee pain and patellar hypomobility. One study [74] provided restrictions on the QT graft type, while five considered the QT harvest site and promoted specific exercises to target the rectus femoris part of the muscle and gain full knee extension [4, 16, 29, 43, 74]. The rationale provided, when present, for any graft‐specific considerations was largely based on biomechanical and/or clinical studies comparing OKC and CKC exercises with respect to ACL strain or joint laxity. Rationale for graft harvest site considerations, however, was largely based on other clinical guidelines, anecdotal observations or basic biomechanics.

**Table 3:** Graft‐specific restrictions and considerations for exercise‐specific recommendations following primary ACLR.

Phase	Structure under consideration		Rationale	Restriction/consideration	Time frame
From Day 1	Graft/fixation		Limit strain to graft/fixation [4, 7]	Isometric knee extension limited to 90–60 [7]	w. 0 [7] to 4 [5]
				Light resistance only (3–5 kg) during OKC knee extension [4, 7]	w. 0–2 [4]
				OKC knee extension limited to 90–45° with heavier load [7]	w. 0 [7] to 4 [5]
			Protect graft [72]	Limit load (OKC 40–90 [72], CKC 0–30 [72])	w. 0–4 [72]
			Avoid graft laxity [84]	OKC only SAQ (light load) during Phase I (0–6 w) [84]	w. 0–6 [84]
			Allow healing [5, 6, 7]	Avoid strenuous activities [5, 7]	w. 0–4 [5]
				Flexor strengthening delayed [6]	w. 0–6–8 [6]
			Allow tendon‐bone healing [83]	Begin dynamic knee flexion no earlier than w. 8 [83]	w. 0–8 [83]
	Harvest site	BPTB	Avoid anterior knee pain [57]	Avoid overstressing extensor mechanism [57]	w. 0–8 [57]
			Avoid patellar hypomobility [48]	Progressive patellar mobilizations are important [48]	w. 0–4 [48]
		QT	Promote patellar mobility [74]		w. 0–8 [74]
			Promote full knee ext [16, 29, 74]	Isolate quadriceps in TKE [29], quad sets, SLR [74]	w. 0–6 [29] to 8 [74]
		Not specified [9]	Limit patellofemoral stress [48]	Limit angle CKC 0–45–60 [48]	w. 0–4 [48]
		Not specified [8]	Limit PJF stress and pain [8]	Limit load (OKC 40–90 [8], CKC 0–80 [8])	0–4 w [8]
		ST	Allow healing [5, 7]	Avoid strenuous activities [5, 7]	w. 0–4 [5]
			Allow healing [43, 57]	Flexor strengthening delayed [43, 48, 57]	w. 0–6–8 [43, 48, 57]
			Protect harvest site [43]	Begin isometric knee flexion no earlier than w. 6 [43]	w. 0–6 [43]
			Protect harvest site [4, 43]	Begin dynamic knee flexion no earlier than w. 8 [1, 43]	w. 0–8 [4, 43]
			Protect harvest site [4, 83]	Delay plyometrics [4, 83]	w. 0–16 [4, 83]
			Promote strength recovery [5]	Hamstring isometric/concentric low intensity at short‐medium muscle length [5]	w. 0–4 [5]
		Not specified [9]	Protect harvest site [57]	Hams strengthening limited [48, 57]	w. 0–4 [48], 0–8 [57]
From w. 2 to 4	Graft/fixation		Graft integrity [40]	Eccentric quadriceps strength [40]	From w. 3 [7]
		QT	Graft fixation [74]	OKC 90–45 [74]	w. 3–4 (B‐QT) [74], w. 3–10 (S‐QT) [74]
	Harvest site	BPTB	For PFJ comfort [4, 43]	Keep knee angle at 45–60 during isometrics [4, 43, 48]	From w. 2 to 4 [43]
		QT	Promote loading/remodeling [4, 16, 43]	Perform knee extension with hip extended [4, 16, 43], stretching [16]	From w. 2 to 4 [43]
From w. 4	Graft/fixation		Avoid undue stress on graft [6] but promote reactivation [7]	OKC loaded [7] knee extension limited to 90–45 [5, 6, 7], with incrementally increased weights [7]	w. 4–12 [7]
		B‐QT	Graft fixation [74]	OKC 45‐0 [74]	From w. 4 to 6 [74]
		BPTB	Avoid graft elongation [80]	OKC knee extension 90–45 [80]	From w. 4 [80]
		ST	Avoid graft elongation [80]	OKC knee flexion no load [80]	w. 4–12 [80]
	Harvest site	ST	Allow healing, limit pain, but promote reactivation [5, 7]	Only low‐intensity isolated hamstring loading [5, 7]	From end of early stage [5]
From w. 6	Graft/fixation		Risk of lengthening [57]	Avoid impact loading [57]	w. 6–12 [57]
From w. 8	Harvest site	ST	Protect harvest site [4, 43]	Dynamic knee flexion with load and pain free 0–90 [4, 43]	w. 8–12 [4, 43]
		QT	Promote loading/remodeling [74]	Perform knee extension with hip extended [74]	w. 8–16 [74]
From w. 10	Graft/fixation	S‐QT	Graft fixation [74]	OKC 45–0 [74]	From w. 10 to 12 [74]
From w. 16	Harvest site	ST	Protect harvest site [4, 43]	Avoid plyometrics [4], but no restriction for other hamstring training [4, 43]	w. 12–16 [4, 43]
		QT	Protect harvest site [74]	Begin plyometrics, but avoid acute spikes [74]	From w. 16 [74]
All phases	Graft	BPTB	Patients will be slower to regain quad strength [4]	Accurate quad strength testing through rehab [4]. Need to identify deficits and modify rehab progression.	Throughout rehab.

Abbreviations: AA, active assisted; ACLR, anterior cruciate ligament reconstruction; BPTB, bone patellar tendon bone; B‐QT, quadriceps tendon with patellar bone block; CKC, closed kinetic chain; LAQ, long arc quadriceps; m, month; OKC, open kinetic chain; PFJ, patellofemoral joint; QT, quadriceps tendon; RM, repetition maximum; ROM, range of motion; RTS, return to sports; SAQ, short arc quadriceps; SLR, straight leg raises; S‐QT, soft‐tissue quadriceps tendon; ST, semitendinosus; TKE, terminal knee extension; w, week.

## DISCUSSION

The most important findings of this scoping review show that the literature on rehabilitation lacks clear and comprehensive graft‐specific considerations when recommending initiation and progression of exercise promoting knee joint loading after ACLR. The 17 publications that included exercise‐based recommendations for rehabilitation after ACLR were mostly Level V evidence (narrative reviews and commentaries) and, notably, only one considered all three graft types.

### Exercise modalities, execution and progression strategies

Using crutches allows immediate, safe (partial) WB and a more symmetrical gait pattern, so this recommendation was an expected finding. Moreover, WB and level ground walking encourage muscle activation and loading of the knee joint through a limited ROM that should not put excessive strain on the graft, its fixation or harvest site. No strong evidence supports the use of a knee brace [1, 70, 84], so it did not come as a surprise that only two papers recommended their use during the early post‐operative phase. Progression to cyclic motion and loading via a stationary bike or stairmaster was often recommended within the first 2–3 weeks, which seems reasonable as loading can be controlled via resistance (bike) and step size and trunk position (stairmaster), considering the individuals' concurrent return to daily activities. Early use of CKC exercises, such as (mini) squatting, offers a way of providing general exercise benefits for all lower limb muscles and joints. However, the relative magnitude of joint moments and contribution of muscle forces through the ankles, knees, and hips depends on the exercise execution, which should be clearly acknowledged within CPGs as a tool to guide and manipulate knee joint loading according to biomechanical principles.

A few of the more recent CPGs recommended a mix of OKC and CKC exercises within the earliest phases of rehab, but often specified a limited (more flexed) ROM to protect the graft from strain close to full extension [20, 45]. Recent SRs, however, indicate that strain values during OKC and CKC exercises are similar, so both types of exercises may be introduced during the early phases to encourage quadriceps activation and terminal knee extension to optimize clinical outcomes [34, 45, 56]. This seems particularly important after QT harvesting, where quadriceps strength deficits are more pronounced [27] and emphasizing hip extended positions are indicated [16]. Negotiating stairs will produce similar strains as an isometric extensor effort at 80% of max at 30° [45], so using short‐ or long‐arc‐quads with light resistance seems reasonable [32, 53]. Notably, recommendations regarding OKC loading were often vague (terms such as ‘light’ or ‘low’ load/intensity) and relied on clinical insight with respect to the patient's ability. Evidence is emerging in support of using blood‐flow restriction to augment low‐load quadriceps strengthening [50], and this modality was often suggested within the extracted papers to allow for effective, early strengthening while limiting strain to the graft and BPTB and QT harvest sites. Neuromuscular electrical stimulation (NMES) for quadriceps strengthening [38, 42] is a more established, recommended modality.

Factors such as type, timing, and dosage of exercises need further investigation, keeping in mind biological aspects such as the harvest sites and likely graft‐dependent differences of osseous integration and ligamentization [56, 58]. The biomechanics of exercise execution is an important consideration during rehabilitation and some recent commentaries do provide clear rehabilitation recommendations based on biomechanical principles and clinical insight [16, 73]. An upright trunk position during squatting should likely be avoided during the earlier phases of rehabilitation as this position will engage the quadriceps with less contribution of hamstrings, resulting in greater strain on the ACL [19]. Moving the trunk forward will shift the contributing joint moment away from the knees to the hips, which lessens quadriceps activity and engages the hamstrings with less strain on the graft [20]. When single‐limb and plyometric exercises are introduced, the interplay of kinematics, kinetics and muscle activation may be of particular importance [28]. This is especially relevant in secondary prevention as the knee valgus moment, a well‐known biomechanical risk factor for ACL injury [71], has a known correlation with relative lateral hamstring versus quadriceps activation during single‐leg landings [28]. The ability of medial hamstrings in countering or mediating valgus stress and anterior shear forces may be compromised after ST harvesting, which makes selective training for augmented semimembranosus activity and hypertrophy particularly relevant.

### Graft‐specific restrictions and considerations

Few exercise considerations focused on protecting the harvest site, and these mainly indicated limited active resisted knee flexion after harvesting from the ST and avoiding hamstring stretching. If hamstring stretches are to be avoided, context should be provided for the performance of OKC extensor exercises, to maintain the trunk reclined during full knee extension, to limit hip flexion during straight leg raises, and the careful use of recumbent stationary bikes. Partial‐thickness harvesting of ST [69] or preservation of the tibial attachment of the sartorius fascia [9] aims to facilitate ST regeneration and improve hamstring outcomes. These are fundamentally different from techniques where complete avulsion of the tendon leaves no continuum of ST tissue, and this should be considered in relation to the timing and magnitude of hamstring activity. Moreover, atrophy and persistent dysfunction of the ST after ACLR is well known [17, 26, 39] and this may create an imbalance in multiplanar knee kinematics/kinetics, despite faster quadriceps strength recovery [27]. Although selective muscle activation of medial versus lateral hamstring may be achieved through strategic exercise [37, 90] and NMES may enhance hamstring strengthening [42], none of the guidelines promoted such selective modalities.

Exercise restrictions after BPTB harvest were few and limited to the first 4–8 weeks addressing patellofemoral joint pain as rationale for limiting load [4, 43, 48, 57]. However, recent studies by Ito et al. [31, 32] using ultrasound imaging indicate that harvesting affects the entire tendon, and that early, targeted loading is warranted to promote healing and patellar tendon hypertrophy for enhanced recovery of quadriceps strength. Their results also showed that healing continues even beyond 8 months after ACLR, which is when many are returning to sports, but no recommendations focused on influencing the healing cascade of the BPTB harvest site, while these were provided after QT harvesting [4, 29, 43, 74] and although regaining quadriceps strength was emphasized by all, only one recommended strength testing throughout rehabilitation [4].

Techniques of harvesting the QT may influence the risk of suprapatellar haematoma and tendinopathy [55, 74] and, thereby, clinical decision‐making. This may impact the safe use of NMES for quadriceps strengthening as the QT harvest site is allowed to heal, but no such restrictions were provided within the extracted guidelines. However, an extended hip during the performance of knee extension was encouraged during the early phase to influence healing and engage the rectus femoris to facilitate full ROM [4, 16, 43]. No restriction was suggested for quadriceps stretching as was done for hamstrings, and one could argue that positioning during hamstring curls (seated vs. prone) might be considered.

Graft revision rates are higher for ST compared to BPTB autografts [76], and MRI studies suggest that BPTB grafts may mature faster than ST [24]. As delayed ligamentization is associated with a greater risk of graft rupture [89], differences in the process of intraosseous integration and ligamentization deserve consideration during rehabilitation. Animal studies show faster bone‐ than tendon‐to‐bone integration after surgery, but this has yet to be supported in human studies [3, 30, 66]. Biologic graft changes in humans are slower than in animals [11], but strategies are available that may enhance healing [23, 78]. Human studies or models indicate that ligamentization involves a 3‐ to 6‐month process of necrosis, revascularization and proliferation [3], followed by remodelling towards maturation, which may occur by 6–12 months [3, 47, 82], but may extend to 2–3 years [11, 88, 89, 91] and is likely influenced by graft type [58]. Systemic loading may promote healing and remodelling of the graft [23, 44], but clinical trials focusing on the influence of timing and magnitude of loading on graft‐specific maturation are needed to improve the evidence base for rehabilitation strategies.

Broad timelines were seen for each phase, suggesting a general approach to rehabilitation, but evidence‐based rehabilitation protocols need to recognize factors such as age, activity level and comorbidities [64], and rehabilitation strategies should align with individuals' characteristics and goals towards optimal function [46, 62]. Performance‐based milestones are important to ensure the necessary prerequisites for more demanding functional tasks, but time‐based criteria should also be used and consider the impact of graft harvesting and surgical technique, although the link between rehabilitation, graft maturation and clinical outcomes is still unknown. Therefore, for optimal translation of research to clinical practice, more high‐quality research is needed to inform initiation, extent and strategies of specific tissue loading after ACLR through detailed exercise descriptors and program variables that consider the surgical procedures [25, 81].

Although efforts were made to reach all relevant literature, some publications may have been omitted due to limitations of the applied search terms and constraints of the specified timeframe and languages.

## CONCLUSION

To support clinical decision‐making, we advocate for the establishment of an international evidence‐based consensus on a graft‐specific approach to rehabilitation, which is sensitive to the implications of exercise execution in terms of moment arms and the muscle agonist/antagonist relationship. This calls for graft‐specific research within biomechanics, utilizing imaging techniques such as MRI and ultrasound to monitor graft and tissue healing, respectively, and adaptation of performance tests and criteria for safe progression.

## AUTHOR CONTRIBUTIONS

Kristín Briem, Mette Kreutzfeldt Zebis and Jesper Bencke conceived of the presented study. All authors developed search terms/strategies. Kristín Briem, Mette Kreutzfeldt Zebis and Linda Fernandes wrote the protocol and conducted the search and review process. Kristín Briem, Mette Kreutzfeldt Zebis, and Linda Fernandes extracted data and prepared it for presentation in tables and figures. All authors contributed to the writing of the manuscript and approved its final version.

## CONFLICT OF INTEREST STATEMENT

The authors declare no conflicts of interest.

## ETHICS STATEMENT

Due to the nature of the research, no ethics approval was needed.

## Supporting information

Supporting information.

## Data Availability

N/A—All relevant data are included in the manuscript.
