# Supplementary material for: Rehabilitation guidelines after autograft anterior cruciate ligament reconstruction need more graft‐specific exercise recommendations—A scoping review
Source: Knee Surg Sports Traumatol Arthrosc. 2025 Apr 3;34(1):83–101. doi: 10.1002/ksa.12666 (PMC12747633; doi:10.1002/ksa.12666)
Supplement: Supplementary file 1 — Supporting information. [file KSA-34-83-s001.docx]

SEARCH STRATEGY FOR SCOPING REVIEW

# **PUBMED: search by MeSH-terms and via title and abstracts (tiab):**

1. **MeSH-terms:**

"Anterior Cruciate Ligament Reconstruction"[Mesh]

"Exercise Therapy"[Mesh] OR "rehabilitation" [Subheading] OR "Rehabilitation"[Mesh] OR "Physical Therapy Specialty/methods"[Mesh]

"Practice Guideline" [Publication Type] OR "Practice Guidelines as Topic"[Mesh] OR "Decision Support Systems, Clinical/standards"[Mesh]

1. **tiab (variations (by using truncation*) and synonyms of the terms, within Titles and abstracts and author keywords):**

“Anterior Cruciate Ligament Reconstructi*” [tiab]

"Exercise Therapy"[tiab] OR rehabilitati* [tiab] OR “Physical Therap*” [tiab]

"guideline*" [tiab] OR "recommend*"[tiab] OR “manage*” [tiab] OR “clinical practice” [tiab]

1. **Resulting search block (combining a) and b) as three search strings, each string using “OR” between the related terms), with the fourth string combining the three search strings, using the “AND” command between strings one through three:**

**#1 Anterior cruciate ligament reconstruction**

"Anterior Cruciate Ligament Reconstruction"[Mesh] OR “Anterior Cruciate Ligament Reconstructi*”[tiab]

**#2 Rehabilitation**

"Exercise Therapy"[Mesh] OR "rehabilitation"[Subheading] OR "Rehabilitation"[Mesh] OR "Physical Therapy Specialty/methods"[Mesh] OR "Exercise Therapy"[tiab] OR “rehabilitati*”[tiab] OR “Physical Therap*”[tiab]

**#3 Guidelines**

"Practice Guideline"[Publication Type] OR "Practice Guidelines as Topic"[Mesh] OR "Decision Support Systems, Clinical/standards"[Mesh] OR "guideline*"[tiab] OR "recommend*"[tiab] OR “manage*”[tiab] OR “clinical practice”[tiab]

**#4**

#1 AND #2 AND #3

# **Web of Science: search via “Topic” (which uses title, abstract, author keywords, and Keywords plus):**

**#1 Anterior cruciate ligament reconstruction**

TOPIC: "anterior cruciate ligament reconstruct*" OR “ACL reconstructi*”

**#2 Rehabilitation**

TOPIC: "exercise therapy" OR rehabilitati* OR "physical therap*" OR physiotherap*

**#3 Guidelines**

TOPIC: "practice guideline" OR "clinical guideline*" OR recommend* OR manage* OR “clinical practice”

**#4**

#1 AND #2 AND #3

# **EBSCO-host (CINAHL Complete (*nursing and allied health*)), using the database’s suggested subject terms for each of the three main topics (in line with the other searches).**

**(MH "Anterior Cruciate Ligament Reconstruction") AND (physical therapy OR physiotherapy OR rehabilitation or exercises) AND (guidelines OR protocols OR practice guideline OR clinical practice guideline OR recommendation)**
